# Editorial Comment: Nocturia and Obstructive Sleep Apnea in Spinal Cord Injured Patients - a Cohort Study

**DOI:** 10.1590/S1677-5538.IBJU.2025.9903

**Published:** 2025-01-05

**Authors:** C Lambert, J Di Maria, P Denys, A Even, A Welniarz, S Hartley, H Prigent, A Leotard, Charles Joussain

**Affiliations:** 1 Centre Hospitalier Universitaire de Bordeaux Service de Médecine Physique et Réadaptation Bordeaux France Service de Médecine Physique et Réadaptation, Centre Hospitalier Universitaire de Bordeaux, Bordeaux, France; 2 AP-HP, Hôpital Raymond Poincaré Neuro-Uro-Andrology R. Poincare Academic Hospital Garches France GHU Paris Saclay, Neuro-Uro-Andrology R. Poincare Academic Hospital, AP-HP, Hôpital Raymond Poincaré, Garches, France; 3 AP-HP, Hôpital Raymond Poincaré Service de Physiologie et d’Explorations Fonctionnelles Garches France Service de Physiologie et d’Explorations Fonctionnelles, GHU Paris Saclay, AP-HP, Hôpital Raymond Poincaré, 104 Boulevard Raymond Poincaré, Garches, 92380, France; 4 UVSQ-Université Paris-Saclay Versailles France UVSQ-Université Paris-Saclay, Versailles, 78000, France; 5 AP-HP, Hôpital Raymond Poincaré Neuro-Uro-Andrology R. Poincare Academic Hospital Garches France GHU Paris Saclay, Neuro-Uro-Andrology R. Poincare Academic Hospital, AP-HP, Hôpital Raymond Poincaré, Garches, France; 6 UVSQ-Université Paris-Saclay Versailles France UVSQ-Université Paris-Saclay, Versailles, 78000, France

**World J Urol. 2024 Sep 11;42(1):519.**

DOI: 10.1007/s00345-024-05190-z|ACCESS: 39259389

## COMMENT

This study intended to describe the prevalence of nocturia and obstructive sleep apnea (OSA) in a cohort of spinal cord injured (SCI) patients and to explore their connection. A retrospective data analysis was undertaken in a tertiary care rehabilitation hospital with specialist sleep and neuro-urology units. All adult SCI patients referred to urodynamic evaluation prior to polysomnography (PSG) between 2015 and 2023 were eligible. Subjective (nocturia) and objective data (urodynamics, polysomnography, built-in CPAP software) were evaluated. Among the 173 patients included, 57.5% had nocturia and 61.9% had OSA. However, research did not discover a statistical link between nocturia and OSA in these individuals. It also revealed significant differences between patients with and without nocturia in terms of neurogenic detrusor overactivity (NDO), volume at first detrusor contraction, and bladder functional capacity, implying that these factors may play an important role in SCI patients with nocturia (1).

The authors concluded that, while both conditions were highly prevalent in SCI patients, there was no direct statistical association between nocturia and OSA in this cohort. Some limitations should be addressed, such as the study's retrospective methodology and the significance of NDO in nocturia in SCI patients. Well-designed prospective studies are still required to better understand the influence of OSA on lower urinary tract symptoms in SCI patients. The effect of bladder overdistension at night has been previously related to the worsening of bladder dysfunction, as well as the occurrence of recurrent urinary infections in this group of patients.
